# Chemical composition and enzyme inhibition of *Phytolacca dioica* L. seeds extracts

**DOI:** 10.1080/14756366.2018.1563077

**Published:** 2019-01-27

**Authors:** Amalia Di Petrillo, Ana María González-Paramás, Antonella Rosa, Valeria Ruggiero, Fabio Boylan, Amit Kumar, Francesca Pintus, Celestino Santos-Buelga, Antonella Fais, Benedetta Era

**Affiliations:** a Department of Life and Environmental Sciences, University of Cagliari, Monserrato, Italy;; b Polyphenols Research Group. Unit of Nutrition and Bromatology, Faculty of Pharmacy, University of Salamanca, Salamanca, Spain;; c Department of Biomedical Sciences, University of Cagliari, Monserrato, Italy;; d Department of Medical Sciences and Public Health, University of Cagliari, Monserrato, Italy;; e School of Pharmacy and Pharmaceutical Sciences, Trinity Biomedical Sciences Institute, Trinity College Dublin, Dublin, Ireland;; f Department of Mechanical, Chemical and Materials Engineering, University of Cagliari, Cagliari, Italy

**Keywords:** *Phytolacca dioica*, tyrosinase, xanthine oxidase, enzyme inhibition, fatty acids

## Abstract

*Phytolacca*, which belongs to the family of Phytolaccaceae, are known for their use in popular medicine. Bioactivity of five extracts from *Phytolacca dioica* seeds were evaluated in four bioassays. A selected group of compounds from the extract that displayed the best bioactivity was analysed. The ethyl acetate extract (EAE) possessed the highest content of phenolics, the highest inhibitory activity on the tyrosinase and xanthine oxidase enzymes and showed a high antioxidant activity. HPLC-DAD-MS was employed to identify the phenolics profile of the most active one (EAE). HSCCC analysis of the EAE led to the isolation of phytolaccoside B and a mixture of 4 isomers, isoamericanol B1, B2, C1 and C2. These isoamericanol isomers presented activity against tyrosinase and xanthine oxidase. Our results revealed for the first time an interesting biological activity of the extract and isolated compounds from *P. dioica* seeds, which could be considered as a source of bioactive molecules.

## Introduction

In the last years, the evaluation of medicinal plants and the isolation of their bioactive compounds have substantially increased due to their potential use as drugs in modern medicine.

Different pharmacologically active compounds from medicinal plants, which can improve health, may act individually, additively or in synergy^1^^,^^2^.

The plant kingdom offers a considerable amount of plants with potential as drug candidates. Species of the genus *Phytolacca,* family Phytolaccaceae, are known for their use in popular medicine. Several ailments have been treated using these plants and different pharmacological actions have been reported, including antimicrobial, anti-inflammatory and anticancer^3–6^. Ethnopharmacological information also revealed that *Phytolacca dioica* is used to heal skin wounds^7^.

Phytochemical screening of the extracts of *P. dioica* leaves and berries revealed the presence of alkaloids, tannins, saponins, phenols, lectins, and flavonoids, while triterpenoid aglycones and phlobatanins were absent^8^. The leaves and berries of *P. dioica* are rich sources of triterpene saponins, which have been described as displaying important biological actions such as molluscicidal, anti-inflammatory, antifungal, and antibacterial activities^9–11^. Moreover, *Phytolacca* species also constitute one of the best sources of ribosome-inactivating proteins (RIPs) that have been used both in the therapy against virus and tumours and in the construction of transgenic plants endowed with resistance to virus, bacteria, fungi, and insects^12^.

However, limited research papers on medicinal and biological properties of *P. dioica* seeds have been published^13^^,^^14^, and scarce information is available on the chemical composition. Therefore, our aim was to investigate the potential bioactivity of *P. dioica* seeds extracts and relate them with the chemical profile. In general, aiming at the isolation of potential bioactive substances. The antioxidant capacity and the inhibitory activity on tyrosinase and xanthine oxidase were evaluated to understand the potential applications of these seeds in medicine.

Indeed, enzyme inhibition is a promising strategy for drug development in several diseases^15–17^. Tyrosinase (E.C. 1.14.18.1) is an enzyme involved in melanogenesis in the skin, leading to the formation of melanin molecules, which act as a protective barrier against UV radiation from the sunlight. The skin is an organ quite vulnerable to oxidative stress and its continuous exposure to direct UV can cause hyperpigmentation and pre-matured aging. Tyrosinase inhibitors are clinically useful for the treatment of some dermatological disorders associated with melanin hyperpigmentation. It has been reported that melanogenesis produces hydrogen peroxide and other reactive oxygen species (ROS) that expose the human melanocytes to high levels of oxidative stress^18^. Thus, inhibitors of melanogenesis could also act as a natural antioxidant in skin care products.

An important biological source of oxygen-derived free radicals is xanthine oxidase (E.C. 1.2.3.2) (XO) that contributes to oxidative damage of living tissues that are involved in many pathological processes^19^^,^^20^. XO catalyses the conversion of hypoxanthine to xanthine and xanthine to uric acid with concomitant production of hydrogen peroxide and superoxide anion. The generation of excess uric acid is harmful to the human body and may lead to gout, hyperuricaemia, and other symptoms of related diseases. Several studies of plant extracts and synthetic compounds have been evaluated for their inhibitory and antioxidant activities in treatment of gout^21^^,^^22^. Therefore, inhibitors of XO may be potentially useful for the treatment of gout or other XO-induced diseases.

Therefore, the goal of our study was to report the chemical composition of the *P. dioica* seed extract to find bioactive molecules, which could be useful as potential drug candidates from natural origin.

## Materials and methods

### Chemical and reagents

All chemicals were obtained as pure commercial products and used without further purification. Standards of fatty acids and fatty acid methyl esters, Desferal (deferoxamine mesylate salt), Trolox, Folin-Ciocalteau’s phenol reagent, 2,2'-azino-bis(3-ethylbenzothiazoline-6-sulphonic acid (ABTS), 2,2-diphenyl-1-picrylhydrazyl (DPPH), kojic acid, allopurinol, XO from cow’s milk, xanthine, and all solvents used, of the highest available purity, were from Sigma-Aldrich (Milan, Italy). The methanolic HCl (3 N) was purchased from Supelco (Bellefonte, PA).

### Plant material

The fruits of *Phytolacca dioica L.* were collected in Cagliari, Italy (coordinates were 39.224195 N, 9.105899 E). The plant was identified by Dr. Cecilia Loi, Department of Life and Environmental Sciences, Section of Botany, University of Cagliari, Italy. A voucher specimen (1233/A Herbarium CAG) has been deposited in the Life and Environmental Sciences Department.

### Preparation of the extracts

Freshly collected samples were washed with running water to remove glochids and impurities, air-dried and hand-peeled. The seeds were separated from the juicy pulp, washed abundantly with distilled water, then dried at room temperature for 24 h, weighed and reduced to a fine powder using a blender type A11 basic (IKA, Germany). The powdered seeds samples were stored at −20 °C prior to analysis.

The powdered seeds (20 g) were extracted in 70% ethanol (ethanol extract, EE). for 24 h at room temperature under continuous stirring. The EE was filtered and centrifuged at 12,000 *g* for 20 min and then evaporated under reduced pressure to dryness. This extract was suspended in distilled water (water extract, WE) and sequentially fractionated by hexane (hexane extract, HE), ethyl acetate (ethyl acetate extract, EAE) and *n*-butanol (butanol extract, BE). After separation of the phases, the solvents were removed in a rotary evaporator at 45 °C under vacuum. All extracts and fractions were submitted to biological assays. All analyses were performed in triplicate.

### Determination of fatty acids

Aliquots of dried HE extract (3 mg) were dissolved in ethanol and subjected to mild saponification at room temperature in the dark^23^. The saponifiable fraction with free fatty acids was collected and the solvent was evaporated. A portion of the dried residue was dissolved in CH_3_CN with 0.14% CH_3_COOH (v/v) and aliquots of the samples were injected into the HPLC system. An aliquot of dried fatty acids was methylated with 1 ml of methanolic HCl (3 N)^23^^,^^24^ for 30 min at room temperature. After addition of *n*-hexane and H_2_O, samples were centrifuged at 900 g. The hexane phase with fatty acid methyl esters was collected, the solvent was evaporated, the residue was dissolved in *n*-hexane and aliquots of the samples were injected into the GC system. All solvent evaporation was performed under vacuum.

### HPLC analysis

Analyses of fatty acids were carried out with an Agilent Technologies 1100 liquid chromatograph (Agilent Technologies, Palo Alto, CA) equipped with a diode array detector (DAD). Analyses of unsaturated free fatty acids (detected at 200 nm), obtained from oil saponification, were carried out with a XDB–C18 Eclipse (150 × 4.6 mm, 3.5 μm particle size) (Agilent Technologies) equipped with a Zorbax XDB-C18 Eclipse (12.5 × 4.6 mm, 5 μm particle size) guard column (Agilent Technologies), with a mobile phase of CH_3_CN/H_2_O/CH_3_COOH (75/25/0.12, v/v/v), at a flow rate of 2.3 ml/min^23^. The temperature of the column was maintained at 37 °C.

Recording and integration of the chromatogram data was carried out through an Agilent OpenLAB Chromatography data system. The identification of fatty acids was made using standard compounds and conventional UV spectra. Calibration curves of all the compounds were constructed using standards and were found to be linear with correlation coefficients > 0.995.

### GC analysis

Fatty acid methyl esters were analysed on a gas chromatograph Hewlett-Packard HP-6890 (Hewlett-Packard, Palo Alto, USA) with a flame ionisation detector (FID) and equipped with a cyanopropyl methyl-polysiloxane HP-23 FAME column (30 m × 0.32 mm × 0.25 μm) (Hewlett-Packard). Nitrogen was used as carrier gas at a flow rate of 2 ml/min. The oven temperature was set at 175 °C, the injector temperature at 250 °C, and the detector temperature at 300 °C. The fatty acid methyl esters were identified by comparison of the retention times to those of standard compounds. The composition of individual fatty acid was calculated as a percentage of the total amount of fatty acids (g %), using the Hewlett-Packard A.05.02 software.

### Determination of total phenolics and flavonoid contents

The total phenolics and flavonoids content were evaluated according to the procedure previously reported^25^^,^^26^. For the total phenolics content, gallic acid was used as the standard, and the results were expressed as milligram of gallic acid equivalents per g of extract (mg GAE/g). Quantification of flavonoids was instead done based on standard curve of quercetin prepared in 80% ethanol and results were expressed in milligram quercetin equivalent per gram of seeds extracts (mg QE/g).

### HPLC-DAD-ESI/MS analysis of phenolic compounds

The EAE of *P. dioica* seeds was analysed using a Hewlett-Packard 1200 chromatograph (Agilent Technologies, Waldbronn, Germany) equipped with a binary pump and a diode array detector (DAD) coupled to an HP Chem Station (rev. A.05.04) data-processing station. The HPLC system was connected via the DAD cell outlet to an API 3200 Qtrap (Applied Biosystems, Darmstadt, Germany) mass spectrometer (MS) consisting of an ESI source and a triple quadrupole-ion trap mass analyser, which was controlled by the Analyst 5.1 software. An Aqua C18 125 Å column (250 × 4.6 mm, 5 μm; Phenomenex) thermostated at 35 °C was used. The solvents were: (A) 0.1% formic acid and (B) acetonitrile. The elution gradient was the same as previously described^27^. Compound identification was made based on their absorption and mass spectral characteristics (both positive and negative modes) and comparison with previously published data.

### HSCCC analysis

High-speed counter-current chromatography (HSCCC) was carried out to isolate bioactive compounds from EAE. HSCCC was performed on Quattro Intro-Prep counter-current chromatograph (AECS, Bridgend, United Kingdom). The choice of solvent system was decided after comparing two-phase solvent systems containing *n*-hexane, ethyl acetate, methanol, and water (H:E:M:W) in different proportions (3:5:3:5 and 1:4:2:3 (v/v/v/v)). The partition coefficients (K_D_) of the compounds in the specific solvent system were evaluated by a UV detector at 254 nm in a Spectroline CX-20 UV Fluorescence Analysis Cabinet, followed by spraying TLC plates with vanillin (2% in methanol) and sulphuric acid (1% in methanol). The system HEMW 1:4:2:3 (v/v/v/v) was chosen because it showed a better distribution of the compounds in the two-phase solvent.

The lower aqueous phase was used as the stationary phase, and the upper organic phase as the mobile phase. The sample solution was prepared by dissolving 500 mg of the dried EAE into 6 ml of two-phase solvent, filtered through 0.45 µm membrane filter prior to injection into the HSCCC system. The column was first filled with the lower stationary phase, subsequently the apparatus was rotated at 850 rpm while the upper phase was pumped into the inlet of the column as the mobile phase at a flow rate of 2 mg/mL. After that equilibrium was established in the column, 6 ml of the extract were injected. After 100 min the rotation was stopped, and the lower phase was pumped into the column and separation was carried out for 100 min more. The fractions were manually collected. The machine was used in the tail to head mode all the time.

The purity of fractions obtained by HSCCC was determined by TLC analysis and HPLC-DAD-ESI/MS analyses and the compounds were identified by MS and 1H NMR analysis^28^.

### Antioxidant assay

Radical scavenging activities were measured by using DPPH and ABTS radical scavenging assays as previously reported^27^. For both free radical methods, antioxidant activity was expressed as concentration of the extract necessary to give a 50% reduction in the original absorbance (half maximal effective concentration, EC_50_).

### Tyrosinase assay

The methodology of the spectrophotometric tyrosinase assay was described in detail in our previous works^27^. The percentage of inhibition of tyrosinase activity was calculated as inhibition (%)=(A − B)/A × 100, where A represents the difference in the absorbance of control sample between an incubation time of 0.5 and 1.0 min, and B represents the difference in absorbance of the test sample between an incubation time of 0.5 and 1.0 min.

The IC_50_ value, a concentration giving 50% inhibition of tyrosinase activity, was determined by interpolation of dose-response curves. Kojic acid was used as a standard inhibitor. The mode of inhibition on the enzyme was performed using the Lineweaver–Burk plot.

### Xanthine oxidase assay

XO activity was determined spectrophotometrically by measuring the formation of uric acid from xanthine. The xanthine solution was prepared by initially dissolving xanthine in a minimal volume of NaOH, adjusting pH to 7.5. The XO solution was prepared by diluting to a final concentration of 0.5 U/ml in cold 0.1 M phosphate buffer (pH 7.5). The assay mixture consisted of 200 μL of plant extract solution, 690 μL 0.1 M phosphate buffer (pH 7.5), 60 μL of xanthine solution and 50 μL of XO. The change in absorbance was recorded at 295 nm for 3 min at room temperature.

Allopurinol was used as a standard inhibitor. Xanthine oxidase activity was expressed as percent inhibition of xanthine oxidase, calculated as (A − B)/A × 100, where A is the change in absorbance of the assay without the plant extract, and B is the change in absorbance of the assay with the plant extract. All assays were performed in triplicate.

The IC_50_ value, a concentration giving 50% inhibition of XO activity, was determined by interpolation of dose-response curves. The mode of inhibition on the enzyme was performed using the Lineweaver–Burk plot. Different concentrations of substrate (20–70 µM) were used for the assay.

### Statistical analysis

Data are expressed as mean ± SD from three independent experiments. The analysis average of the treatment using multiple comparisons was determined by using Duncan’s multiple range tests, and the data were compared using the *p* values: *p* < .05 was considered statistically significant. The least significant difference (LSD) was used to determine the difference between the methods used to the investigation of the various antioxidant capacities. The statistical analysis of differences between various treatments was determined by the Student’s *t*-test. Values of *p* < .05 were considered statistically significant. Statistical analysis was performed with SPSS v.18.0 (IBM Corp., Armonk, NY, USA).

## Results and discussion

### Analyses of fatty acids

To give some insight into the chemical composition of *P. dioica* seeds, quali-quantitative information on the individual fatty acids in HE extracts was obtained by GC and HPLC-DAD analyses. Fatty acid compositions (expressed as % of total fatty acids, g/100 g) of oil obtained by GC analysis are reported in Table 1. HE showed a concentration of approximately 20.82 ± 0.03% of saturated fatty acids (SFA, mainly palmitic acid 16:0, and stearic acid 18:0, being about 16.6 and 2% respectively), 51.71 ± 3.25% of monounsaturated (MUFA, mainly oleic acid 18:1 n-9, 50.3%), and 16.0 ± 1.1% of polyunsaturated (PUFA), mainly constituted by linoleic 18:2 n-6, 15.3%.

**Table 1.** Fatty acid composition (%, g/100 g) by GC analysis of *P. dioica* HE.

Fatty acid	g/100 g
12:0	0.57 ± 0.14
14:0	0.93 ± 0.45
16:0	16.58 ± 0.63
16:1 *n-7*	0.20 ± 0.07
18:0	1.96 ± 0.18
18:1 *n-7*	0.20 ± 0.07
18:1 *n-9*	50.32 ± 3.43
18:2 *n-6*	15.33 ± 1.01
18:3 *n-3*	0.67 ± 0.08
20:0	0.77 ± 0.11
20:1 *n-9*	0.91 ± 0.19
SFA	20.82 ± 0.03
MUFA	51.71 ± 3.25
PUFA	16.00 ± 1.10

SFA: saturated fatty acids; MUFA: monounsaturated fatty acids; PUFA: polyunsaturated fatty acids.

Mean and standard deviation of 3 samples (*n* = 3).

Furthermore, the absolute content of the main unsaturated fatty acids in the *P. dioica* HE was determined by HPLC, as follows: approximately 488.7 mg/g of oil extract for 18:1 n-9, and minor amounts for 18:2 n-6 and 18:3 n-3 (148.4, and 8.1 mg/g of oil extract, respectively).

MUFA formed a main part of fatty acid composition and the majority fatty acid identified in *P. dioica* oil extract was oleic acid (18:1 n-9).

### Total phenolics and flavonoids content

The total phenolics content of the samples was quantified in each *P. dioica* seeds extract (Table 2). The EAE extract exhibited the highest phenolics content. In fact, it was respectively 2.8 and 3.9 fold higher than that determined in BE and EE extracts. The EAE also yielded the highest amount of flavonoids, followed by WE, EE, and BE. As it was expected, HE was the poorest in both phenolic and flavonoid compounds. Previous studies have reported the total phenolic and flavonoid contents for other species of Phytolacca^29^^,^^30^. However, to best of our knowledge no studies have investigated the phenolic and flavonoid contents for the *P. dioica* seeds. It is important to highlight that the total phenolic and flavonoid contents reported in this study are appreciably higher than the previous studies^29^^,^^30^.

**Table 2.** Total phenolics and flavonoid content and free radical scavenging activity of *P. dioica* extracts

Extract	Total phenolics^(*)^	Flavonoids^(**)^	EC_50_ values (µg/mL)	
			ABTS scavenging	DPPH scavenging
EE	102.07 ± 2.30^c^	18.43 ± 1.22^bc^	16.97 ± 0.32^a^	20.4 ± 1.23^a^
HE	2.61 ± 0.21^a^	1.82 ± 0.42^a^	>500	>500
EAE	396.41 ± 4.21^e^	43.57 ± 1.26^d^	3.1 ± 0.04^a^	4.85 ± 0.11^a^
BE	143.64 ± 1.35^d^	15.19 ± 0.54^b^	5.64 ± 0.26^a^	6.28 ± 0.91^a^
WE	46.34 ± 0.42^b^	19.66 ± 0.39^c^	127.48 ± 8.37^b^	137.79 ± 5.53^b^
Mixture of isoamericanol B1, B2, C1 and C2	–	–	7.1 ± 0.4	6.51 ± 0.74
Phytolaccoside B	–	–	>500	>500
Trolox	–	–	3.4 ± 0.3	3.2 ± 0.4

(*) mg GAE/g of dry weight.

(**) mg QE/g of dry weight.

The data are given as mean ± standard deviation (SD) of triplicate experiments. The statistical comparison between values from the different plant extracts was performed using the *post hoc* Duncan test. Means followed by distinct letters in the same column were found to be significantly different (*p* < .05).

### HPLC-DAD-ESI/MS analyses of phenolic compounds

**Figure 1.**
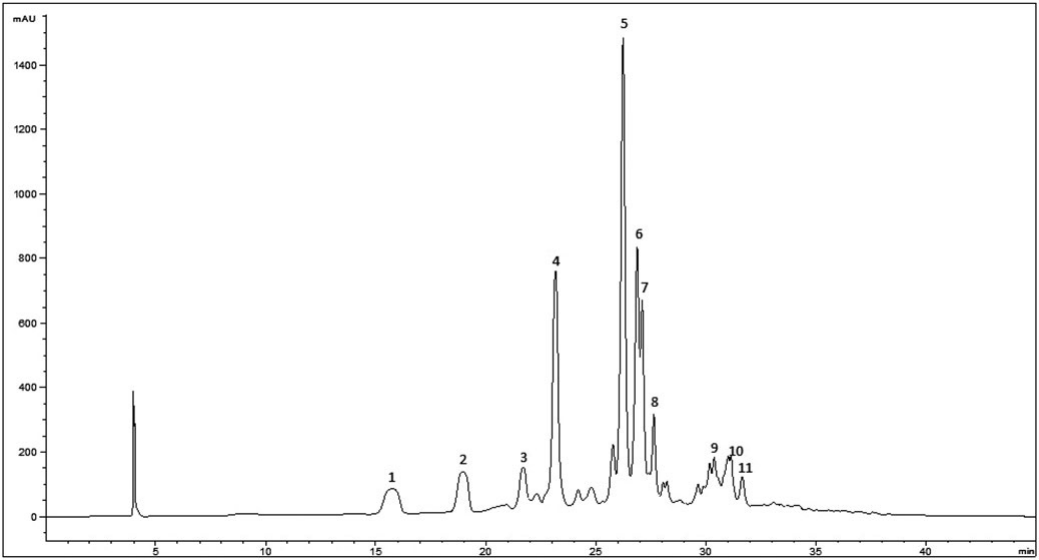
HPLC chromatogram of P. dioica seeds ethyl acetate extract recorded at 330 nm for phenolic compounds. Peak identification is given in Table 3.

To identify the nature of phenolic compounds contained in the EAE of *P. dioica* seeds, HPLC-DAD-ESI/MS analyses were carried out. A representative HPLC chromatogram recorded at 330 nm is shown in Figure 1, and the phenolics’ composition is summarised in Table 3.

**Table 3.** Identification of polyphenol compounds in *P. dioica* seeds ethyl acetate extract by HPLC-DAD-ESI/MS analysis.

Peak	Rt (min)	*λ*_max_ (nm)	Pseudomolecular ion [M-H]^−^ (*m/z*)	MS^2^ (*m/z*), (%)	Tentative identification
1	15.7	342	163	135(100)	p-coumaric acid
				121(42)	
				145(41)	
				119(20)	
2	19	330	329	137(97)	(iso)americanol A
3	21.7	324	297	135(41)	caffeoyl-threonic acid (isomer I)
				179(23)	
4	23.1	320	687	179(100)	Unknown
				133(100)	
				269(28)	
5	26.2	334	493	329(100)	isoamericanol B1 or related isomers (B2 / C1 / C2 / (iso)princepin)
				327(100)	
				165(64)	
				175(35)	
				137(32)	
6	26.8	338	493	165(100)	isoamericanol B1 or isomers (B2 / C1 / C2)
				298(75)	
				329(40)	
				147(23)	
7	27.1	320	297	135(36)	caffeoyl-threonic acid (isomer II)
				179(16)	
8	27.6	320	462	289(93)	Unknown lignan
				165(100)	
				147(32)	
9	30.3	334	657	493(100)	Unknown lignan
				327(41)	
				165(25)	
10	31.1	324	329	211(32)	trihydroxy-octadecenoic acid
				229(31)	
				171(18)	

Relevant peaks in the extract were assigned as lignans. Peaks 5 and 6 were tentatively identified as different isomers of isoamericanol (B1, B2, C1 or C2) with the molecular mass of 494^31^. This identification was further supported by the MS^2^ fragments at *m/z* 329 that might correspond to (iso)americanol A (also associated to peak 2) and at *m/z* 327 to (iso)americanin A^31^^,^^32^.

### HSCCC and structural identification of isolated compounds

Two compounds were isolated from EAE extract using HSCCC. Structure of purified compounds was elucidated by interpretation of spectroscopic experiments (UV, MS, and ^1^H NMR) and by comparison with literature.

Compound **1**: Mixture of Isoamericanol B1, B2, C1, and C2 UV (MeOH) *λ*_max_ nm: 334; HRESI–MS *m/z* [M − H]+: 493 (related to isoamericanol C1 and C2); ^1^H NMR (Methanol-D4, 400 MHz)^28^ (Table 4).

**Table 4.** NMR compound 1 – mixture of isoamericanol B1, B2, C1 and C2

Isoamericanol (B1, B2, C1, C2)	^1^H-NMR	^13^C-NMR
1	–	129.2
2	6.90 (d, 2.5 Hz)	115.9
3	–	146.2
4	–	146.3
5	6.83 (d, 8.5 Hz)	115.7
6	6.76 (dd, 2.5, 8.5 Hz)	119.4
7	4.80 (d, 8 Hz)	76.2
8	3.97 (m)	78.7
9	3.46 (dd, 12.5, 5.5)	60.6
	3.65 (dd, 12.5, 3)	
1’	–	130.7
2’	7.00 (m)	117.2
3’	–	145.7
4’	–	145.7
5’	6.94 (m)	119.2
6’	6.94 (m)	119.8
7’	–	76.1
8’	3.97 (m)	78.6
9’	3.46 (dd, 12.5, 5.5)	60.6
1”	3.65 (dd, 12.5, 3)	131.0
2”	6.94 (m)	115.9
3”	–	144.0
4”	–	145.6
5”	6.90 (d, 2.5)	117.5
6”	4.17 (dd, 6, 1.5 Hz)	119.2
7”	6.47 (d, 16 Hz)	129.8
8”	6.17 (dt, 16.6, 7.8 Hz)	128.5
9”	–	62.0

Compound **2**: UV (MeOH)*λ*_max_(MeOH)nm: 210; HRESI–MSm/z[M − H]^−^: 6 6 5 [M + H] +, 5 1 5 [M + H-150]+;^1^H NMR (Methanol-D4, 400 MHz): 0.78 (3H, s, H26), 0.91 (3H, s, H29), 1.12 (3H, s, H27), 1.15 (3H, s, H24), 1.26 (3H, s, H25), 2.68 (2H, brd, H-18), 3.66 (3H, s, COOMe), 4.35 (1H, d, J = 8 Hz, H3), 5.31 (1H, brs, H12), 5.32 (1H, d, 8 Hz, H1 Xyl). Compound **2** was identified as phytolaccoside B (Table 4).

### Radical scavenging activity

*P. dioica* extracts were evaluated for their antioxidant activity using two different methods, leading to quite similar values for each extract analysed. In agreement with the phenolics content, the HE showed the poorest antioxidant activity, whereas the EAE and BE showed the highest antioxidant activity (Table 2). EC_50_ values of EAE and BE are comparable to that of Trolox, used as reference compound (Table 2). Compound 1 (Mixture of isoamericanol B1, B2, C1 and C2) showed an EC_50_ for ABTS and DPPH of 7.1 and 6.5 µg/mL (Table 2) respectively, explaining in part the antioxidant activity determined in the EAE extract.

The bioassays experiments revealed antioxidant activity of the EAE and its constituent (compound **1**) to be remarkably pronounced with respect to previous studies performed on *Phytolacca americana*^29^^,^^30^. Since, no data has been reported in the literature about the antioxidant activity of *P. dioica* seeds extracts.

### Enzymes inhibitory activity

All extracts were also evaluated for their inhibitory effect on tyrosinase and xanthine oxidase enzymes (Table 5). The obtained results revealed that EAE exhibited the most potent inhibitory activity against mushroom tyrosinase. BE and EE were less potent, and no activity was detected for HE and WE.

**Table 5.** Inhibitory activity of the extracts of *P. dioica* seeds against tyrosinase and xanthine oxidase at 200 µg/mL.

Extract	Inhibitory activity (inhibition % ± SD)	
	Tyrosinase	Xanthine oxidase
EE	22.35 ± 0.49^a^	50.5 ± 0.99^b^
HE	ND	ND
EAE	50.83 ± 0.84^c^	99.93 ± 1.94^c^
BE	29.23 ± 0.90^b^	23.475 ± 1.66^a^
WE	ND	ND
Kojic acid	99.98 ± 0.02	
Allopurinol		99.96 ± 0.003

ND: Not detected.

The results are expressed as Inhibition % (μg/mL). The data are given as mean ± standard deviation (SD) of triplicate experiments. The statistical comparison between values from the different seeds extracts was performed using the *post hoc* Duncan test. Means followed by distinct letters in the same column were found to be significantly different (*p* < .05).

EAE was also the most potent inhibitor of xanthine oxidase among all extracts; it exhibited 99.93 ± 1.94% inhibition at a concentration of 200 µg/mL. EE and BE showed 50.5 ± 0.99% and 23.47 ± 1.66% inhibition, respectively, at same conditions, whereas the remaining extracts (HE and WE) showed no XO activity.

**Figure 2.**
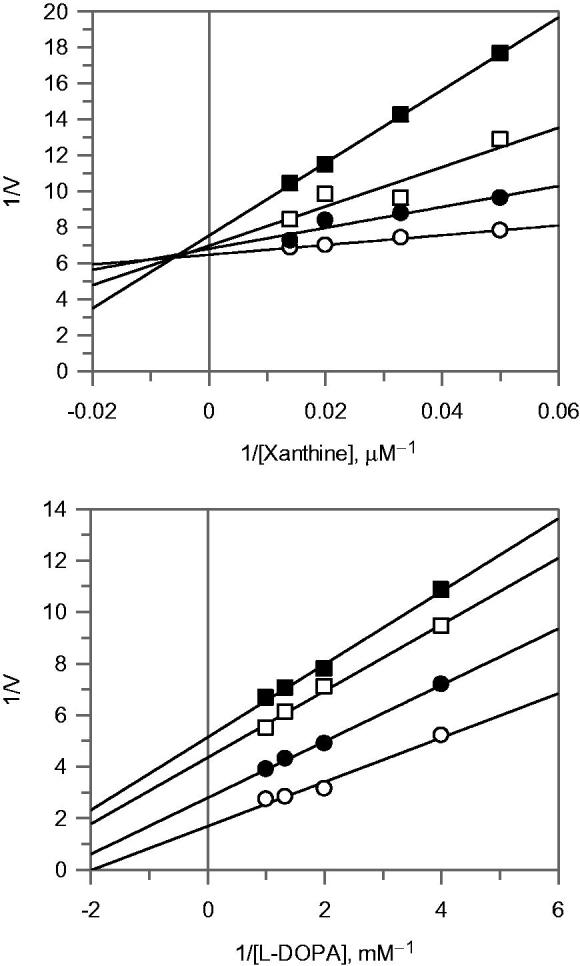
Lineawear-Burk plot for inhibition of EAE extract on xanthine oxidase (A) and tyrosinase (B). The inhibitor concentrations were 0 (○), 0.04 (•), 0.08 (□) and 0.1 (▪) mg/mL, for xanthine oxidase assay. The inhibitor concentrations were 0 (○), 0.05 (•), 0.1 (□) and 0.2 (▪) mg/mL, for tyrosinase assay.

Considering the complete inhibition of XO by EAE, further attention was focussed on its mode of inhibition of the enzyme, which was determined by Lineweaver–Burk plot analysis, as shown in Figure 2(A). The kinetic analysis revealed that this extract acts as a mixed-type inhibitor. In fact, increasing the concentration of the extract resulted in a family of lines, which intersected in the second quadrant. To calculate the equilibrium inhibition constants for the inhibitor binding with the free enzyme (K_I_) and the enzyme-substrate complex (K_IS_), two secondary graphs (data not shown) were obtained by plotting the *K*_m_/*V*_max_ (slope) and 1/*V*_max_ (intercept) versus the concentration of EAE. The values of *K*_I_ and *K*_IS_ were determined to be 0.68 and 0.007 mg/mL respectively.

The mode of inhibition of this extract on tyrosinase was also determined using Lineweaver–Burk plot which displayed an uncompetitive inhibition of the enzyme, see Figure 2(B). The equilibrium constant for binding with enzyme–substrate complex (*K*_IS_*)* was calculated to be 0.113 mg/mL.

The isolated mixture of isoamericanol B1, B2, C1, and C2 showed inhibitory activity on tyrosinase and xanthine oxidase enzymes, with IC_50_ of 0.110 ± 0.02 mg/mL and 0.145 ± 0.05 mg/mL respectively. No activity was detected for the phytolaccoside B.

### Molecular and physicochemical properties of compounds

Physicochemical properties of three classes of isoamericanol were calculated using Swiss-ADME web tool^33^ (Table 6). Due to critical importance for pharmacokinetics drug discovery, we report, in addiction to simple molecular attribute, the lipophilicity, water solubility, and drug likeliness characteristics for the three classes. Among the three classes of isoamericanol, A and B display similar molecular flexibility reflected from their same number on rotatable bonds, while C1 exhibiting highest flexibility. Further, isoamericanol C1 possess the highest number of hydrogen (H-bond) acceptor and donor atoms, molecular refractivity and polar surface area (PSA). On the other hand, isoamericanol B showed highest lipophilic behaviour, while best water solubility characteristics was noted for isoamericanol A.

**Table 6.** Molecular properties of three classes of isoamericanol compounds.

Physicochemical properties	Isoamericanol A	Isoamericanol B	Isoamericanol C1
Molecular formula	C18H18O6	C19H20O5	C27H26O9
Molecular Weight (g/mol)	330.33	328.36	494.49
Rotatable bonds	4	4	6
H-bond acceptor atoms	6	5	9
H-bond donor atoms	4	2	5
Molar refractivity	87.87	91.18	129.20
Polar Surface área (Å^2^)	99.38	68.15	138.07
Lipophilicity (consensus)	1.65	2.82	2.24
Water solubility	Soluble	Moderate	Moderate

Pharmacokinetics	Isoamericanol A	Isoamericanol B	Isoamericanol C1
Gastrointestinal absoprtion	High	High	High
Blood brain barrier permeation	No	Yes	No
P-glycoprotein substrate	Yes	Yes	No
Cytochrome P450 2D6 inihibitor	No	Yes	No
Cytochrome P450 3A4 inihibitor	No	No	Yes
Druglikeness (lipinsky rule)	Yes	Yes	Yes

**Figure 3.**
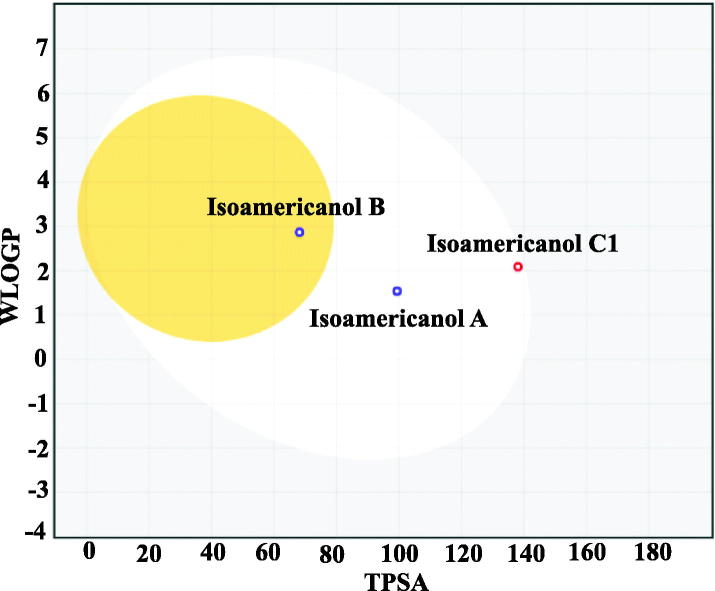
Novel BOILED-Egg construction using two physicochemical descriptors, lipophilicity (LogP) and polar surface area (PSA) for the three isoamericanol compounds. The white region represents the physicochemical space of gastrointestinal absorption and the yellow region brain penetration.

Concerning pharmacokinetics, all the three classes displayed high gastrointestinal absorption, while only isoamericanol B showed promising blood brain barrier (BBB) permeation characteristics. We used a novel Egg-boiled model, which is simple and intuitive graph prediction of passive intestinal absorption and brain penetration, as a function of lipophilicity and apparent polarity (Figure 3). If plotted molecule falls inside the white ellipse, the probability of a good intestinal absorption is high, while inside the yellow ellipse (i.e. the yolk), the probability of a good BBB crossing is high. Interestingly, all the three classes displayed drug likeness calculated according to Lipsinky’s rule.

## Conclusions

The development of new inhibitors, synthetic or from natural sources, of tyrosinase and xanthine oxidase activity is a very promising field of research^34–37^.

The results of this study showed that the ethyl acetate extract of *P. dioica* seeds possessed a good XO and tyrosinase inhibitory activity.

Xanthine oxidase catalyses the oxidation of hypoxanthine to xanthine and subsequently to uric acid. This enzyme plays a vital role in the onset of hyperuricaemia and gout. Furthermore, its re-oxidation involves molecular oxygen and during this reaction reactive oxygen species are produced. The obtained results indicate that the ethyl acetate extract of *P. dioica* seeds presents constituents with medicinal properties that could be exploited to treat diseases associated with oxidative stress and xanthine oxidase enzyme activity. This extract can also be considered a good candidate for further investigations to evaluate its effect on the inhibition of skin pigmentation as deduced from its effects on tyrosinase.

Moreover, the isolated mixture of isoamericanol (B1, B2, C1, and C2) showed inhibitory activity on tyrosinase and xanthine oxidase enzymes. This is the first time that these isoamericanols have been described as inhibitors of the above mentioned enzymes and the presence of these compounds in the ethyl acetate extract may contribute to the activity observed for the plant. Indeed, we found promising physicochemical and pharmacokinetics properties for the three classes of isoamericanol.

The ethyl acetate extract also showed highest antioxidant capacity if compared with other extracts. The EC_50_ values were strongly correlated with total phenolic contents, suggesting the contribution of these compounds to the antioxidant properties of the extracts. Moreover, most of the polyphenolics identified were lignans with well-known antioxidant activity^38^.

Furthermore, the chemical composition of the hexane extract from the *P. dioica* seeds showed that major fatty acid identified was oleic acid (18:1 n-9), which is very important for the nutritional value of oils. It has been documented that MUFA may reduce LDL cholesterol, while it might possibly increase HDL cholesterol^39^. Oleic acid may promote insulin resistance and it has been reported as anti-apoptotic and anti-inflammatory agent via down regulation of cyclooxygenase-2 and inducible nitric oxide synthase through the activation of nuclear factor-kappa B (NF-κB)^39^.

All in all, for the first time the chemical composition and inhibitory enzyme activity of *P. dioica* seeds extracts is here reported. It seems that this plant could be a source of bioactive molecules. The results achieved open promising perspectives for further studies of the biological effect and medicinal use of these natural products.
